# Revealing mechanism of Methazolamide for treatment of ankylosing spondylitis based on network pharmacology and GSEA

**DOI:** 10.1038/s41598-023-42721-x

**Published:** 2023-09-16

**Authors:** Tao Sun, Manzhi Wang, Weiqiang Liang, Ping Gao, Qiang Liu, Xinfeng Yan

**Affiliations:** 1 Department of Orthopedic Surgery, The First Affiliated Hospital of Shandong First Medical University & Shandong Provincial Qianfoshan Hospital, No. 16766, Jingshi Road, Lixia District, Jinan City, Shandong Province China; 2 Department of Hematology, The First Affiliated Hospital of Shandong First Medical University & Shandong Provincial Qianfoshan Hospital, Jinan, Shandong China; 3 Department of Cardiology, Shandong Medicine and Health Key Laboratory of Cardiac Electrophysiology and Arrhythmia, The First Affiliated Hospital of Shandong First Medical University & Shandong Provincial Qianfoshan Hospital, Shandong First Medical University, Jinan, Shandong China

**Keywords:** Pharmacology, Target identification

## Abstract

Methazolamide is a carbonic anhydrase (CA) inhibitor with satisfactory safety. Our previous studies have demonstrated the elevation of CA1 expression and the therapeutic effect of Methazolamide in Ankylosing spondylitis (AS). In this study, we explored the pathogenic role of CA1 and the pharmacological mechanism of Methazolamide in AS through Gene Set Enrichment Analysis (GSEA) and network pharmacology. Seven out of twelve CA1 related gene sets were enriched in AS group. CA1 was core enriched in above seven gene sets involving zinc ion binding, arylesterase activity and one carbon metabolic process. Functional analysis of the candidate target genes obtained from the intersection of AS associated genes and Methazolamide target genes indicated that Methazolamide exerts therapeutic effects on AS mainly through inflammatory pathways which regulate the production of tumor necrosis factor, IL-6 and nitric oxide. PTGS2, ESR1, GSK3β, JAK2, NOS2 and CA1 were selected as therapeutic targets of Methazolamide in AS. Molecular docking and molecular dynamics simulations were performed successfully. In addition, we innovatively obtained the intersection of Gene Ontology (GO)/Kyoto Encyclopedia of Genes and Genomes (KEGG) analyses and GSEA results, and found that 18 GO terms and 5 KEGG terms were indicated in the pharmacological mechanism of Methazolamide in AS, involving bone mineralization, angiogenesis, inflammation, and chemokine signaling pathways. Nevertheless, validation for these mechanisms is needed in vivo/vitro experiments.

## Introduction

Ankylosing spondylitis (AS) is considered as a chronic inflammatory disorder that mainly affects the axial skeleton^1^, and leads to the bony fusion of vertebral joints in its late stage^2^. Patients with AS usually need arthroplasty in their end stage^3,4^. Clinical manifestations of AS include inflammatory back pain, sacroiliitis, enthesitis, and involvement of specific organs accompanied with HLA-B27 positivity in majority patients^4,5^. Inflammatory and autoimmune pathways are mainly involved in the pathogenesis of AS^6^. However, common pharmacological treatments for AS, including nonsteroidal anti-inflammatory drugs (NSAIDs), disease-modifying antirheumatic drugs, analgesics, topical glucocorticoids, and biologics such as tumor necrosis factor (TNF) inhibitors and interleukin-17 (IL-17) /IL-23 monoclonal antibodies, only alleviated inflammation but showed limited effect on retarding radiological progression of AS^7–10^. In addition, these treatments are associated with heavy economic burden and many adverse effects such as infection, osteoporosis and cardiovascular or gastrointestinal effects after long-term application^11,12^.

Apart from inflammation, ectopic new bone formation is another pathological feature of AS^13^, which leads to the bony fusion of vertebral joints in an extreme form. Recent studies have demonstrated the role of classical osteogenic pathways including Wnt/β-catenin signalling^14,15^, BMP/Smads signalling^16,17^ and hedgehog signalling^18,19^ in ectopic new bone formation at the late stage of AS. However, therapeutic treatment focusing on new bone formation is still lacking. Carbonic anhydrases (CAs) are a large family of zinc metalloenzymes which catalyze the reversible hydration of carbon dioxide. CA1 participates in the formation of calcium carbonate^20^, and leads to abnormal bone mineralization and ossification by competitively decreasing the production of hydroxyapatite^21,22^. Furthermore, the obvious higher arthritic score, thicker hind paws and more proportion of collagen II induced arthritis were observed by Zheng Y and his colleagues in transgenic mice over-expressing CA1^23^. In our previous study, we have found a significant elevation of CA1 expression in the synovium of AS patients compared with the samples of rheumatoid arthritis and osteoarthritis patients^24^, which indicated that CA1 participated in the unique pathogenic mechanism of AS. In addition, we have confirmed that Methazolamide, a CA inhibitor, could ameliorate both Bath Ankylosing Spondylitis Disease Activity Index (BASDAI) and Bath Ankylosing Spondylitis Functional Index (BASFI) assessments of AS patients with satisfactory safety. Methazolamide also contributed to the clearer articular surfaces of sacroiliac joints in AS patients^25^. Therefore, we speculated that CA1 may be a novel pathogenic factor in AS, and Methazolamide exhibited a safe and effective therapeutic performance in AS patients.

In order to further explore the pharmacological mechanism of Methazolamide and the pathogenic mechanisms of CA1 in AS, we used network pharmacology and Gene Set Enrichment Analysis (GSEA) in this study.

## Materials and methods

### Identification of AS associated genes and Methazolamide target genes

We searched GeneCards database (https://www.genecards.org)^26^, Online Mendelian Inheritance in Man database (OMIM, https://www.omim.org)^27^ and Therapeutic target database (TTD, http://db.idrblab.net/ttd/)^28^ for genes with specific query terms “Ankylosing spondylitis”. The union of OMIM genes, TTD genes and the highest quartile of genes obtained from GeneCards was selected as AS associated genes. Human target genes of Methazolamide were obtained from the union of SwissTargetPrediction database (http://www.swisstargetprediction.ch)^29^ and TargetNet database (http://targetnet.scbdd.com)^30^. The Methazolamide target genes were filtered by studies performed in Homo sapiens. We took the intersection of AS associated genes and Methazolamide target genes by Venny 2.1 (https://bioinfogp.cnb.csic.es/tools/venny/index.html) and got the candidate target genes of Methazolamide in AS.

### Protein–protein interaction (PPI) network construction

Based on the identified candidate target genes, PPI networks were constructed using the Search Tool for the Retrieval of Interacting Genes Database (STRING)^31^ (https://cn.string-db.org/) and visualized by Cytoscape software (version 3.7.2)^32^. Because of the limited number of candidate target genes, confidence scores were set to low values in order to include more genes (like CA1) in the network (confidence scores > 0.15). CytoHubba, a plug-in for Cytoscape software that measures nodes according to topologic analysis and therefore allows exploring important nodes in biological networks^33^, was used to identify hub genes. Since the amount of candidate target genes was relatively small, we selected the top 5 hub genes and CA1 for following molecular docking.

### Functional annotation and pathway enrichment analysis

To further reveal the functions of candidate target genes, Gene Ontology (GO) annotation and Kyoto Encyclopedia of Genes and Genomes (KEGG) pathway enrichment analysis of candidate target genes were performed using the ‘cluster profiler’ package in R software^34^. GO terms consist of the following three components: biological process (BP), cellular component (CC) and molecular function (MF). GO functional enrichment analysis and KEGG pathway enrichment of the significant module were conducted using the Database for Annotation, Visualization, and Integrated Discovery (DAVID) 6.8^35^ (http://david.abcc.ncifcrf.gov/) online tool. A P value < 0.05 was set as the cutoff criterion. And bubble plot of KEGG results was illustrated by ggplot2 and Hmisc packages in R.

### Prediction of binding models between Methazolamide and candidate target proteins

The 3D structure of Methazolamide was downloaded from Pubchem website (https://pubchem.ncbi.nlm.nih.gov)^36^, and was convert to a file with pdbqt type using Open Babel (version 2.4.0)^37^ and PyMOL (version 2.5). The crystal structures of target proteins (PTGS2 (PDB:5F1A), ESR1 (PDB:1X7E), GSK3β (PDB:1Q3W), JAK2 (PDB:3KRR), NOS2 (PDB:4CX7) and CA1 (PDB:3LXE)) were downloaded from the RCSB Protein Data Bank (https://www.rcsb.org/)^38^ and were modified using PyMOL and Autodock 4.2 software^39^ to remove ligands and water, and add hydrogen and charges. Molecular docking was performed using Autodock Vina (version 1.2.0)^40^. The best docking models were recognized with the highest affinity and the smallest root mean square deviation (RMSD) between the predicted conformation and the observed X-ray crystallographic conformation. Models with RMSD ≤ 4 Å were considered reliable and those with RMSD ≤ 2 Å were considered accurate^41^. Finally, the docking complex was analyzed using Protein–Ligand Interaction Profiler (https://plip-tool.biotec.tu-dresden.de/plip-web/plip/index)^42^ and visualized using PyMOL.

### Re-docking for docking validation

The docking protocol was validated by re-docking process. We firstly removed the protein’s co-crystallized ligand (PDB ID: 5F1A). Then the ligand was re-docked into the same place after being added hydrogen and computed gasteiger through Autodock vina. The RMSD was calculated in PYMOL between the co-crystallized ligand and the lowest energy posture achieved by re-docking. The re-docking operation was considered reliable when the RMSD was within the trusted range of 2 Å^43^.

### Molecular dynamics (MD) simulations

For the evaluation of the vibrant binding behavior and binding consistency of protein–ligand complexes in various docked poses, a 100 ns of MD simulation was conducted on GROMACS(2022.1)^44^. The procedure was carried out under constant temperature, constant pressure, and periodic boundary conditions. The proteins were placed in the Amber14SB all-atom force field^45^, while the ligand was placed in the GAFF small molecule force field based on Amber and TIP3P water model^46^. During MD simulation, all bonds involving hydrogen atom were constrained by the LINCS algorithm^47^, with an integration step of 2 fs. The electrostatic interaction was calculated by the Particle mesh Ewald (PME) method^48^. The cut off value of non-bonded interaction was set as 10 Å. The simulated temperature was controlled at 298.15 K by the V-rescale^49^ coupling temperature method. The pressure was controlled at 1 atm by the Parrinello-Rahman method^50^. Firstly, in order to eliminate the close contact between atoms, the steepest descent method was used to minimize the energy of the two systems. Then, molecular simulation was executed at 298.15 K temperature and 1 atm pressure over a 500 ps NVT and NPT production run respectively. Finally, the simulation was extended to 100 ns for all the complexes to analyze their behavior in long run. RMSD value and Root mean square fluctuation (RMSF) value were used to analyze the structural changes of complexes. Also, visualization of molecular dynamics simulations can be seen through VMD^51^.

### GSEA in AS

GSE73754 is the whole blood gene microarray of fifty-two AS patients and twenty healthy controls. Gene expression profiles of GSE73754 were obtained from the Gene Expression Omnibus (GEO) database^52^. The normalized original data (see Supplementary Spreadsheets S1 online) was confirmed by the box-plot. The GO and KEGG gene sets associated with CA1 were download from GSEA database (http://www.gsea-msigdb.org/gsea/)^53^ for further analysis. Source organism was Homo sapiens, and contributors were Gene Ontology Consortium and Kyoto Encyclopedia of Genes and Genomes. Then the normalized expression data of GSE73754 and CA1 related gene sets were uploaded to GSEA 4.2.3 software for analysis^54^. The gene sets from GSEA were excluded when their sizes were less than 5 or larger than 2000. Normalized enrichment score (NES) > 1 and false discovery rate (FDR) < 0.25 were applied to evaluate enrichment magnitude and statistical significance, respectively.

**Figure 1:**
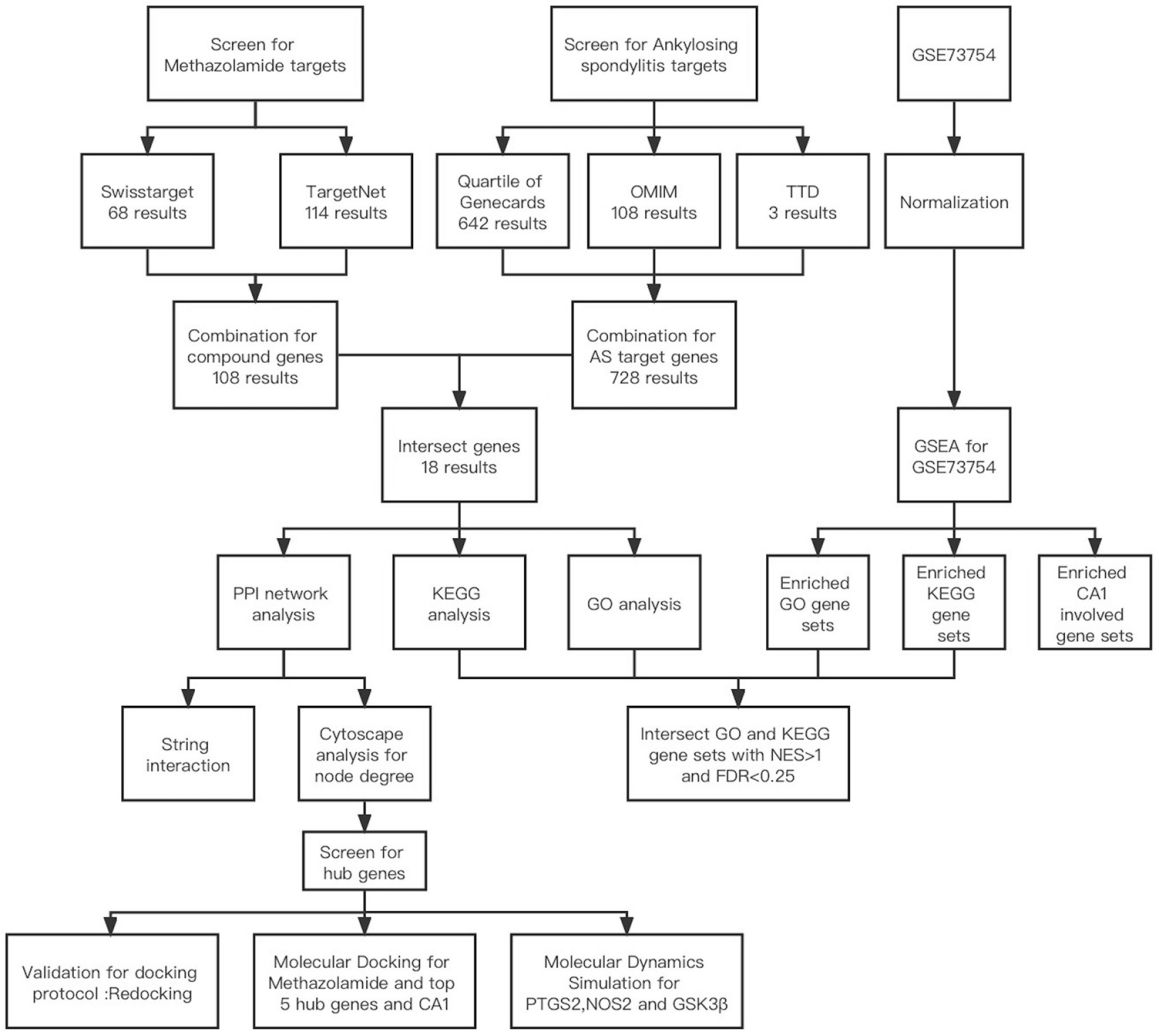
The flowchart for revealing the therapeutic effects of Methazolamide in ankylosing spondylitis. *AS* ankylosing spondylitis, *PPI* protein–protein interaction, *CA1* carbonic anhydrase1, *KEGG* Kyoto Encyclopedia of Genes and Genomes, *GO* Gene Ontology, *OMIM* Online Mendelian Inheritance in Man database, *TTD* Therapeutic target database, *GSEA* Gene Set Enrichment Analysis, *NES* normalized enrichment score, *FDR* false discovery rate.

We further uploaded the normalized expression data of GSE73754 and whole GO (c5.go.v7.5.1.symbols) and KEGG (c2.cp.kegg.v7.5.1.symbols) gene sets to GSEA 4.2.3 software for analysis, and got the AS associated pathway terms. The key GO and KEGG terms were defined as the intersection of AS associated pathway terms and pathway terms associated with candidate target genes mentioned above. The flowchart was shown in Fig. 1.

## Results

### The candidate target genes from the intersection of AS associated genes and Methazolamide target genes

For AS associated genes, there were 3 genes in TTD database, and 108 genes in OMIM database. In GeneCards database, the AS associated genes had extremely lower median score (1.03). Considering both accuracy and completeness, we took the highest quartile of genes from GeneCards database which contained 642 results. The union of the above results was obtained, and totally 728 AS associated genes were selected after removing the duplicates (see Supplementary Spreadsheets S2 online).

For Methazolamide target genes, there were 114 genes in TargetNet database and 68 genes in SwissTargetPrediction database. 14 genes in SwissTargetPrediction database were chosen for further analysis since their probability values were larger than 0. Finally, the union of the above results was obtained, and a total of 108 genes were identified as Methazolamide target genes after removing the duplicates (see Supplementary Spreadsheets S3 online).

There were 18 candidate target genes obtained from the intersection of AS associated genes and Methazolamide target genes: *CA1, ALPL, CTDSP1, DNMT1, ESR1, GPR35, GSK3β, HNF4A, HTR2A, JAK2, MIF, MMP8, NOS2, PTGS1, PTGS2, RELA, RIPK2 and TLR9.*

### PPI network of candidate target genes

**Figure 2:**
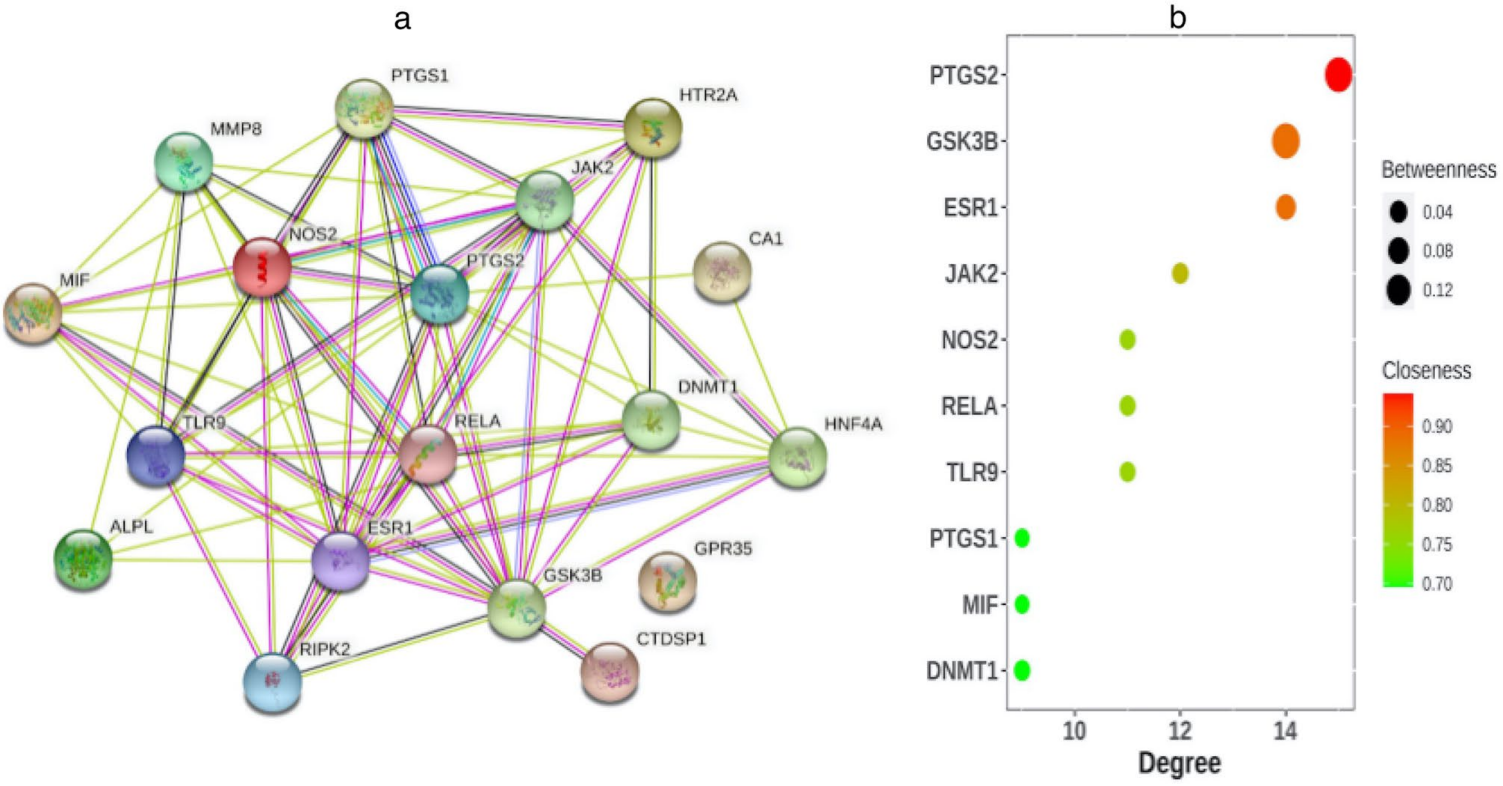
(**a**) The protein–protein interaction (PPI) network of target proteins established by Search Tool for the Retrieval of Interacting Genes (STRING). (**b**) Bubble chart demonstrating the degree, betweenness, and closeness of hub genes. The horizontal axis represents the degrees of genes. The longitudinal axis represents different genes. The size of the bubbles indicates betweenness. The different colors represent closeness.

The PPI network of 18 candidate target genes was gained from STRING (Fig. 2a), including 18 nodes and 75 edges. To explore genes that may play an important role in the pharmacological mechanism of methazolamide in AS, hub genes were identified by CytoHubba. Since biological networks are heterogeneous, multiple topological analysis algorithms (Degree, MCC, MNC and EPC) were used simultaneously to identify hub genes in the PPI network (see Supplementary Spreadsheets S4 online). And the top 10 candidate target genes with degrees larger than the average degree (8.82) were showed in Fig. 2b. The top 6 hub genes from the intersection of four algorithms were selected for further analysis. They are *PTGS2, GSK3β, ESR1, JAK2, TLR9, NOS2.* Among them, the most significant target against AS was *PTGS2* which had the largest values among the four topological algorithms.

### GO and KEGG enrichment analysis of candidate target genes

**Figure 3:**
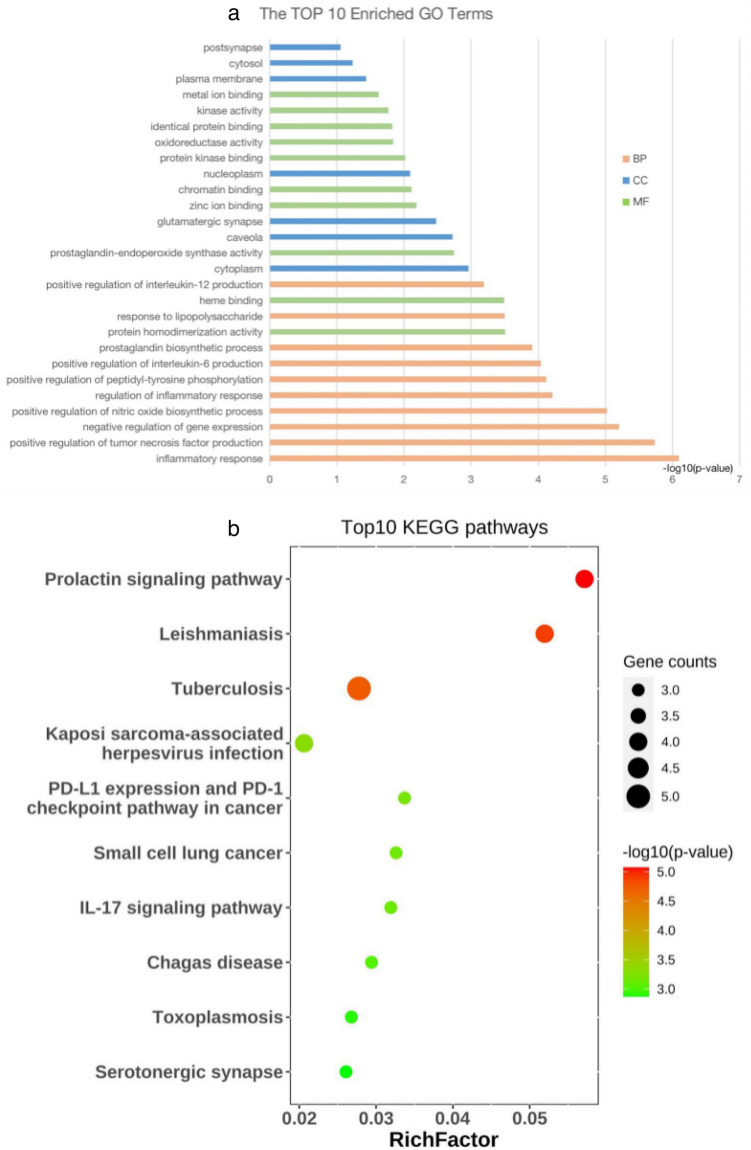
(**a**) Gene Ontology (GO) analysis of target genes. The horizontal axis represents − log10 (p-value) of each term. The longitudinal axis represents different GO terms. The top ten enriched biological process terms (labeled in orange), molecular function terms (labeled in green) and cellular component terms (labeled in blue) were included in the chart. (**b**) The top ten Kyoto Encyclopedia of Genes and Genomes (KEGG) pathways for target genes of Methazolamide in ankylosing spondylitis. The horizontal axis represents RichFactor of each term. The longitudinal axis represents different KEGG terms. The size of the bubbles indicates gene counts. The different colors represent − log10 (p-value).

Totally, the candidate target genes were significantly enriched in 79 GO terms and 16 KEGG terms. In biological process, the enrichments were in inflammatory response, positive regulation of tumor necrosis factor production, negative regulation of gene expression, positive regulation of nitric oxide biosynthetic process, and regulation of inflammatory response. In molecular function, the enrichments were in protein homodimerization activity, heme binding, prostaglandin-endoperoxide synthase activity, zinc ion binding, and chromatin binding. In cellular component, the enrichments were in cytoplasm, caveola, glutamatergic synapse, nucleoplasm, and plasma membrane (Fig. 3a). In KEGG pathway, the candidate target genes were significantly enriched in prolactin signaling pathway, tuberculosis, leishmaniasis, pathways in cancer and Kaposi sarcoma-associated herpesvirus infection (Fig. 3b).

### Molecular docking of Methazolamide and target proteins in AS

**Figure 4:**
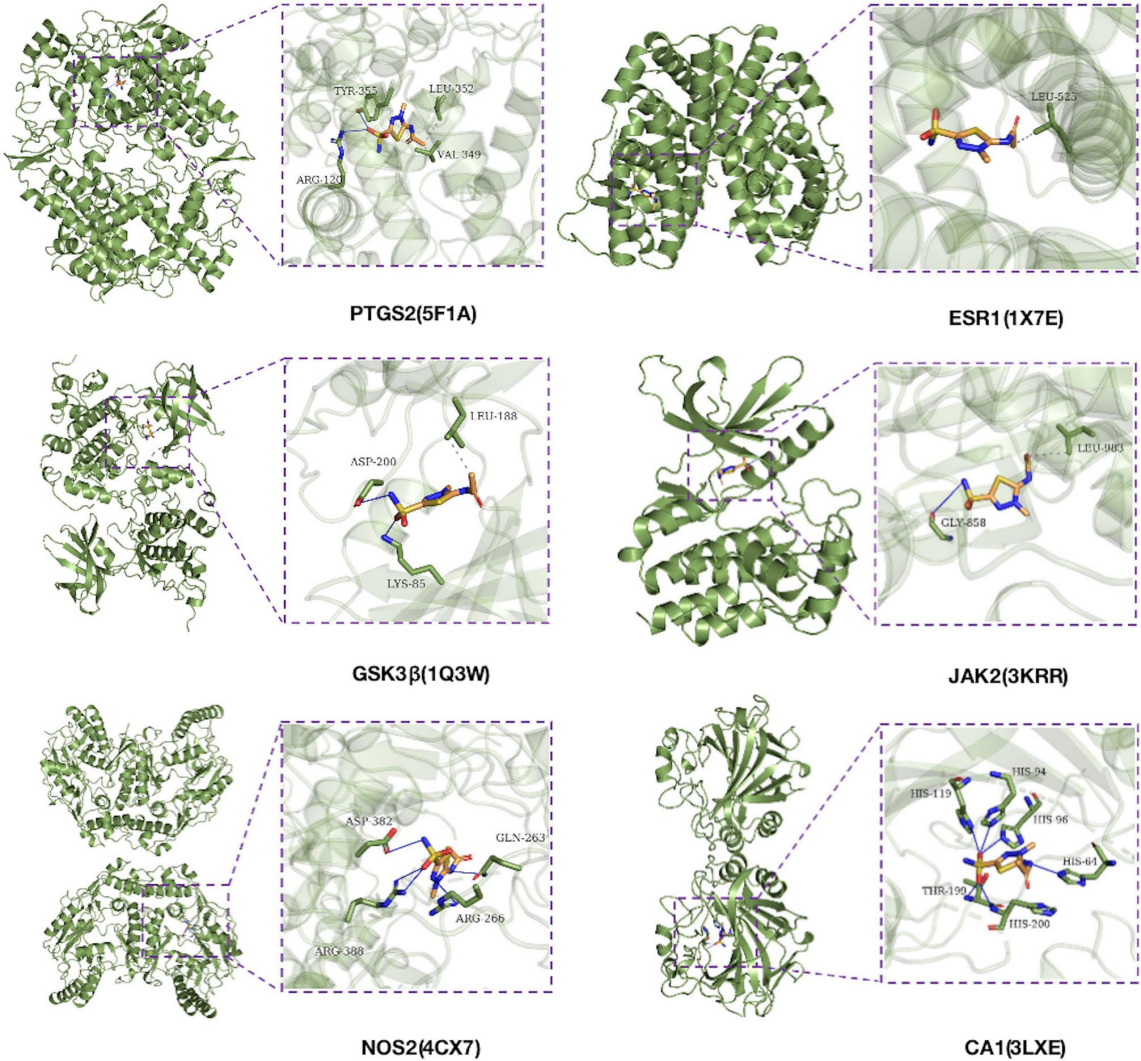
Molecular models of Methazolamide binding to the predicted target proteins. The blue solid lines indicate hydrogen bonds and the blue dotted lines indicate hydrophobic interactions. The binding regions were marked with rectangles.

The top 6 hub genes identified by CytoHubba were *PTGS2, GSK3β, ESR1, JAK2, TLR9, NOS2*. Since we only found the crystal structure of target protein TLR9 from horse origin, the potential target proteins (PTGS2 (PDB:5F1A), ESR1 (PDB:1X7E), GSK3β (PDB:1Q3W), JAK2 (PDB:3KRR) and NOS2 (PDB:4CX7)) of Methazolamide in AS were selected for molecular docking. In addition, as a CA inhibitor, Methazolamide was also docked to CA1(PDB:3LXE). Molecular docking was performed successfully between Methazolamide and six candidate target proteins (Fig. 4).

All the docking predictions were summarized in Supplementary Table S1 online. For instance, Methazolamide was bound to PTGS2 by hydrophobic interactions with VAL-349 (length 3.76 Å) and LEU-352 (3.55 Å), and through forming hydrogen bonds with ARG-120 (DIST_H-A 2.46 Å) and TYR-355 (2.28 Å). Similarly, Methazolamide was predicted to dock into the binding pocket of CA1 via forming hydrogen bonds with HIS-64 (2.11 Å), HIS-94 (2.16 Å), HIS-96 (2.22 Å), HIS-119 (2.48 Å), THR-199 (2.00 Å) and HIS-200 (2.43 Å).

### Re-docking results for docking protocol

**Figure 5:**
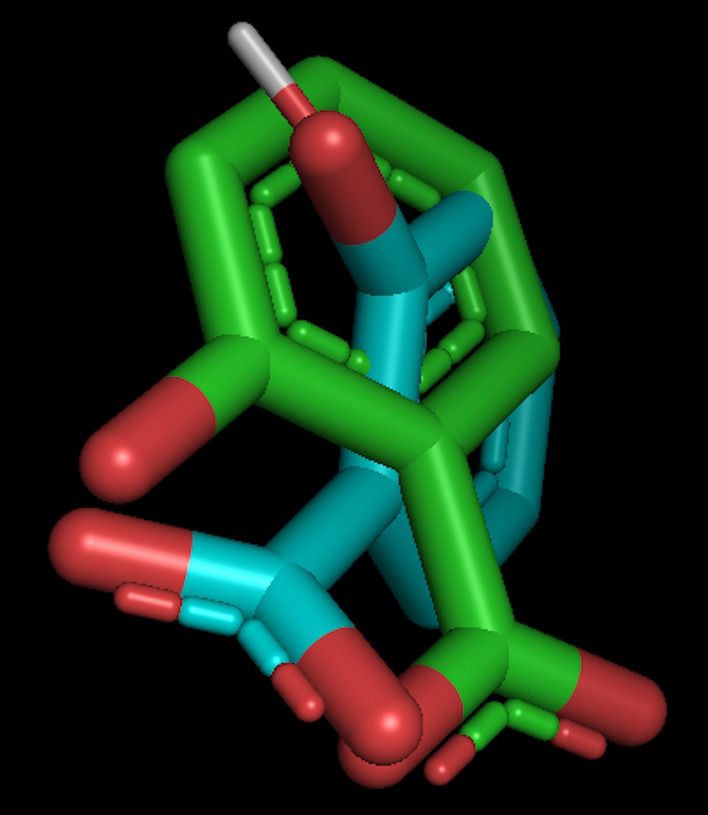
Superimpose view of re-docking RMSD value of 0.821 Å (Green: Original, Blue: Docked) in the active site using PyMOL.

The re-docking of the co-crystallized ligand was applied to validate the correctness of docking algorithms. The 3D superimposition of the co-crystallized ligand and the lowest energy posture achieved by re-docking was shown in Fig. 5, and the RMSD is 0.821 Å. This was helpful to test the docking protocol’s validity and accuracy in part.

### MD simulations

We chose PTGS2, GSK3β and NOS2 complexes for MD simulations, since they had more interactions with Methazolamide in molecular docking.

#### RMSD analysis

**Figure 6:**
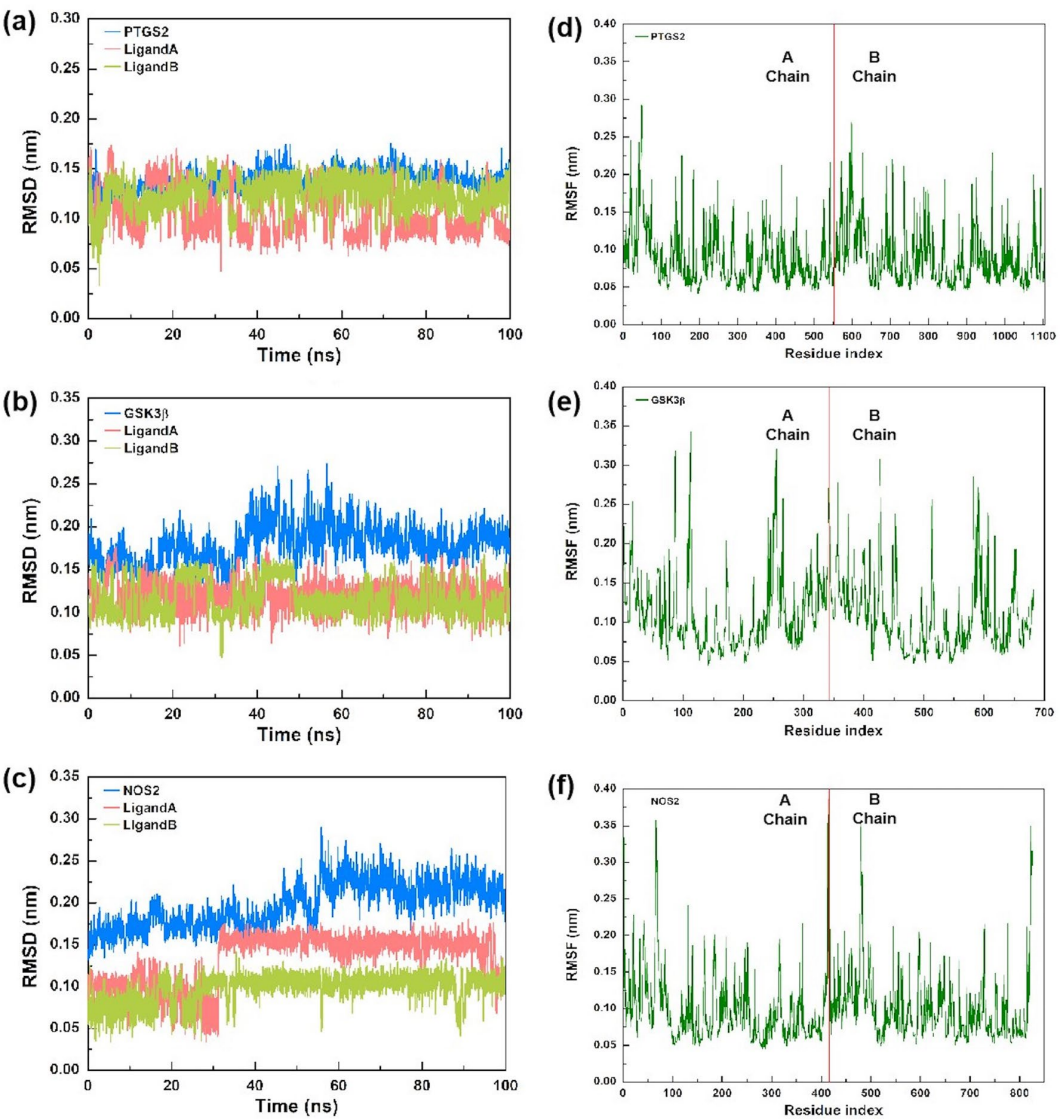
The MD simulation trajectories from 100-ns simulation time, Blue color represents the protein and red or green color represent the ligand, here (**a**) RMSD plot of PTGS2- Methazolamide complex; (**b**) RMSD plot of GSK3β- Methazolamide complex; (**c**) RMSD plot of NOS2- Methazolamide complex; (**d**) RMSF plot of PTGS2- Methazolamide complex; (**e**) RMSF plot of GSK3β- Methazolamide complex and (**f**) RMSF plot of NOS2- Methazolamide complex.

RMSD revealed the predicted conformational changes from the original structure across the simulation period. The fluctuation of RMSD is also an important indicator to determine whether the simulation is stable or not. The RMSD values for three protein–ligand complexes over simulation time (100 ns) were calculated and shown in Fig. 6a–c, respectively. During the simulation, the protein backbones were found to be consistent. And no abnormal deviations of RMSD were found among all three complexes in comparison to the native structures, which indicated that the ligand molecule can stably bind to the active pocket of the proteins without inducing significant impacts on their spatial structures. More precisely, for PTGS2 complex, the system was equilibrated, and the backbone was stable until the end of simulation. The average RMSD values of PTGS2, ligand A and ligand B were 0.138 ± 0.010 nm, 0.107 ± 0.024 nm, and 0.125 ± 0.015 nm respectively. For GSK3β complex, the backbone was also consistent and stable throughout the simulation. The average RMSD values of GSK3β, ligand A and ligand B were 0.179 ± 0.023 nm, 0.121 ± 0.016 nm, and 0.116 ± 0.020 nm respectively. For NOS2 complex, in the case of ligand A, initially the backbone RMSD was consistent until 31 ns; after that, there was a small flip, and then consistency was achieved until the end of simulation. Higher deviation during the above duration might be due to high level of conformational changes. The average RMSD values of NOS2, ligand A and ligand B were 0.197 ± 0.027 nm, 0.133 ± 0.031 nm and 0.098 ± 0.016 nm respectively. Hence, the above observations revealed the stability of three protein–ligand complexes in the dynamic state.

#### RMSF analysis

RMSF measurements were used to indicate structural stability, atomic mobility, and residue flexibility during the simulation. For PTGS2 complex, estimated RMSF values of the whole structure were less than 0.3 nm (Fig. 6d), indicating high stability of the complex. For GSK3β complex, there was a fluctuation more than 0.3 nm at ASP-87, ARG-111, LYS-113, LYS-255, and SER-426, and the remaining structure was stabled comparatively (Fig. 6e).For NOS2 complex, the most fluctuating atoms were the LYS-66, GLN-413, LYS-479, and HIS-823 with 0.3580, 0.3978, 0.3519, and 0.3504 RMSF, respectively(Fig. 6f). Changes in conformation may be the cause of the deviation. The RMSF results further indicated that the ligand molecule can bind to the active pocket and exist stably in all three protein complexes.

### Further verification by GSEA in AS

#### The GSEA results of CA1 in AS

To further identify the pathogenic role of CA1 in AS, we used GSEA to analyze the validate dataset (GSE73754) from the GEO database. Totally, 11 CA1 associated GO gene sets and 1 CA1 associated KEGG gene set were downloaded for further analysis. The dataset GSE73754 contained 23,396 genes which were available for GSEA. The GSEA results showed that seven out of twelve gene sets were enriched in AS group. In biological process, the enrichment was shown in one carbon metabolic process. In molecular function, the enrichments were shown in zinc ion bind, transition metal ion binding, hydrolase activity acting on ester bonds, arylesterase activity and carbonate dehydratase activity. In KEGG pathway, the enrichment was shown in nitrogen metabolism pathway. The NES and FDR of GSEA results were summarized in Table 1.

**Table 1:** The GSEA results for CA1 involved KEGG and GO enriched terms.

CA1 involved KEGG and GO enriched terms	NES	FDR q-val
Zinc ion binding	1.53	0.021
Transition metal ion binding	1.46	0.014
Hydrolase activity acting on ester bonds	1.19	0.190
Arylesterase activity	1.15	0.195
One carbon metabolic process	1.14	0.174
Carbonate dehydratase activity	1.05	0.289
Nitrogen metabolism	1.09	0.360

*GSEA* gene set enrichment analysis, *CA1* carbonic anhydrase1, *KEGG* kyoto encyclopedia of genes and genomes, *GO* gene ontology, *NES* normalized enrichment score, *FDR* false discovery rate.

**Figure 7:**
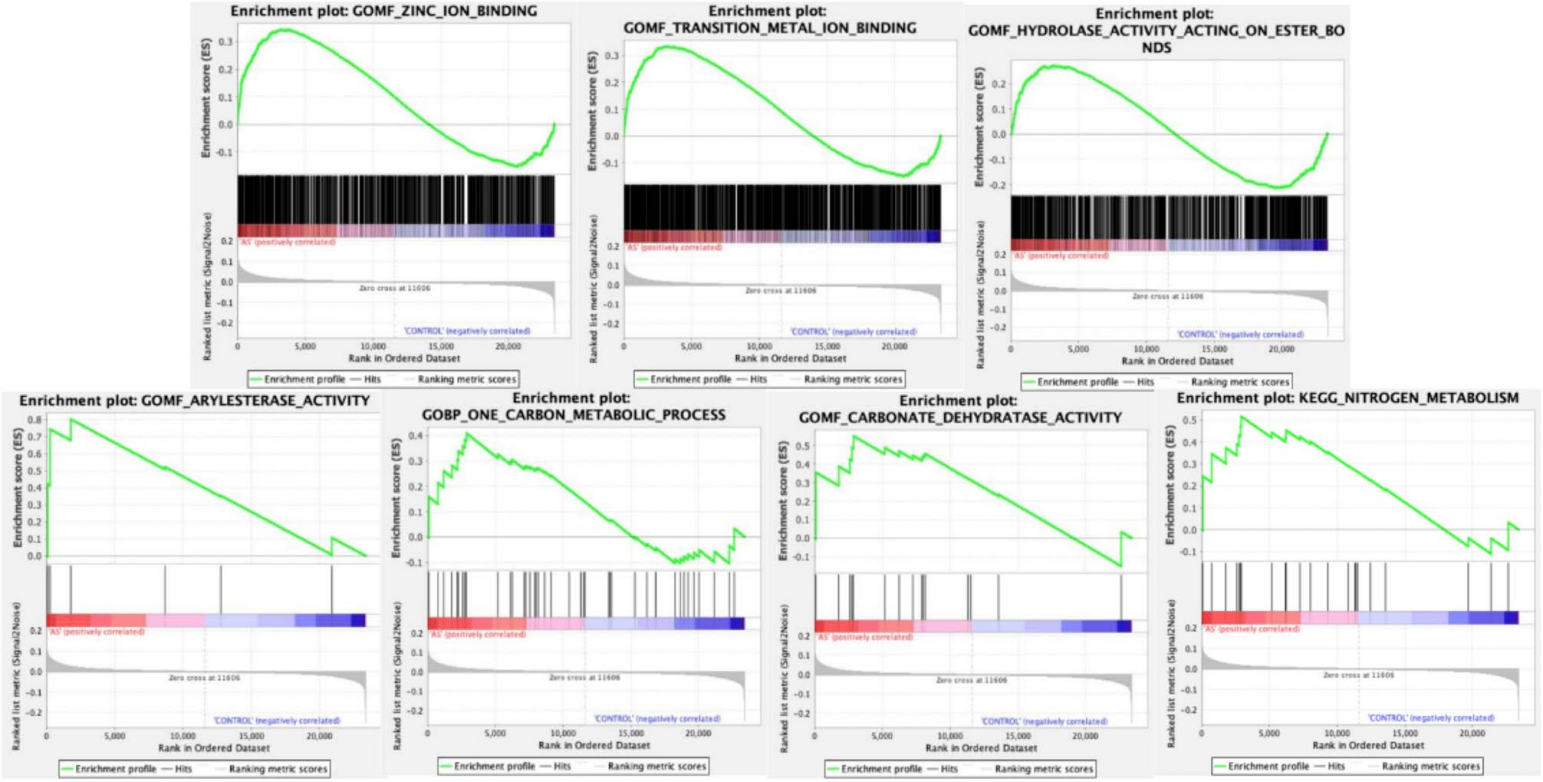
Snapshot of enrichment results for CA1 involved GO and KEGG gene sets in GSE73754.The green line represents Enrichment Score (ES). The black lines represent genes belong to examined gene set. Enrichment gene sets were selected when the peak of ES appeared in AS positively correlated area (red region). *CA1* carbonic anhydrase1, *GO* Gene Ontology, *KEGG* Kyoto Encyclopedia of Genes and Genomes, *AS* ankylosing spondylitis.

CA1 was shown core enrichment in above seven gene sets (Fig. 7).

In addition, CA1 ranked the 101th of 23,396 genes in the AS associated rank ordered gene list (see Supplementary Spreadsheets S5), indicating the critical role of CA1 in the development of AS. Notably, GSEA results also showed that the gene *APOBEC3A*, which was belonged to the target genes of Methazolamide, ranked the 35th of 23,396 genes in the AS associated rank ordered gene list. However, the role of *APOBEC3A* in AS is unclear, and further validation is needed.

#### The intersection of GO/KEGG analyses and GSEA results

To further verify whether the hub candidate genes mentioned above play a critical pathogenic role in AS, we rerunning GSEA with total GO and KEGG gene sets in the validate dataset (GSE73754) from the GEO database. There were 3915 GO gene sets and 102 KEGG gene sets enriched in AS group of GSE73754. All of six hub candidate genes mentioned above were up-regulated in AS group. These GSEA results were further intersected with the significantly enriched GO and KEGG terms of candidate target genes mentioned above. Totally,18 GO terms and 5 KEGG terms were selected, including small cell lung cancer, pathways in cancer, neurotrophin signaling pathway, chemokine signaling pathway in KEGG pathway and regulation of synaptic vesicle exocytosis, positive regulation of jnk cascade, dioxygenase activity, response to vitamin d, transcription coactivator binding, bone mineralization, and negative regulation of protein catabolic process in GO terms. These intersected terms represented the specific pharmacological mechanism of Methazolamide in AS. NES and FDR of the intersected terms were summarized in Supplementary Table S2. And the bold black terms with NES > 1 and FDR < 0.25 should be further studied in future.

## Discussion

Ankylosing spondylitis is a chronic inflammatory rheumatic disease which occurs more frequently among young men and has a hereditary nature^1^. Until now, the pathogenesis of AS is not completely understood. Previous researches mainly focused on the inflammatory pathways^1,4,6^. However, we have noticed that the pathophysiological features of AS comprise bone resorption, bone destruction and new bone formation^1^, and CA1 which was significantly elevated in the synovium of AS patients^24^ may participate in the aberrant osteogenesis process^21–23,55^. It is well known that a layer of hydroxyapatite was formed on the surface of bioactive glasses upon immersion in simulated body fluids, which simulating the normal physiological bone mineralization. Over-expression of CA1 can produce more calcium carbonate which competitively decrease hydroxyapatite production^21,22^, inducing aberrant osteogenesis and further aggravating joint inflammation and tissue destruction^55^.

To explore the specific pathogenic mechanism of CA1 in AS, we chose GSEA for further analysis. The GSEA method derives its power by focusing on gene sets that share common biological function, chromosomal location, or regulation. Compared with single-gene methods, GSEA features a number of advantages such as more reproducible and more interpretable, detecting modest changes in individual genes by boosting the signal-to-noise ratio, and defining functional gene subsets by the leading-edge analysis^54^.

In the present study, the GSEA results indicated that seven CA1 related gene sets were enriched in AS, including zinc ion binding, arylesterase (ARE) activity and one carbon metabolic process. Systemic use of zinc showed positive impact on cartilage repair of osteoarthritis in in vitro and animal studies^56^. Furthermore, hypovitaminosis A caused by the low level of zinc dependent hepatic retinol binding protein synthesis can lead to a markedly decreased antioxidant capacity and enhanced eicosanoid production in RA^57^. Therefore, as a zinc ion binding protein^58^, CA1 may play a pathogenic role in AS by reducing the concentration of zinc ion^59^. Decrease in the ARE activity leads to the generation of oxidative stress and may play an important role in the pathogenesis of AS. Moreover, the activity of ARE in patients with AS is strictly correlated with the activity of the inflammatory process^60^. CA1 may affect the ARE activity by regulating its catalytic enzymes which contain zinc ions^61^. Therefore, ARE may be a link between CA1 and oxidative stress/inflammatory response in the pathogenesis of AS. The term ‘one-carbon metabolism’ refers to a system of interdependent metabolic pathways that facilitate the transfer of the one-carbon units that are needed for DNA methylation, dTMP synthesis, and purine synthesis^62^. Recently discovered novel disease pathways in AS included those involving DNA methylation^63,64^. For example, methylation of the suppression of cytokine signaling 1 (SOCS-1) can be detected in serum of HLA-B27 positive AS patients. Moreover, the methylation of SOCS-1 was significantly associated with the severity of patients’ spondylopathy, sacroiliitis and acute phase reactant C-reactive protein^65^. Therefore, CA1 can regulate AS related gene expression by methylation modification through one-carbon metabolism process. These findings indicated that CA1 is involved in the specific pathogenic mechanism of AS and can be a potential therapeutic target for AS.

Methazolamide is a CA inhibitor. In our previous study, Methazolamide seemed to be a potential therapeutic drug with appropriate validity and safety for AS patients. Considering the interdependence of cellular and molecular participants in biological systems, a drug may have far broader effects than its finite molecular target^66^. Network pharmacology is the novel, promising, cost-effective drug development approach that encompasses systems biology, network analysis, connectivity, redundancy and pleiotropy^67^. In this study, we tried to identify the potential therapeutic targets of Methazolamide in AS through network pharmacology.

First, we obtained the candidate target genes from the intersection of AS associated genes and Methazolamide target genes. Functional analysis of the candidate target genes indicated that Methazolamide plays a therapeutic role in AS mainly through the inflammatory pathways which involve the production of TNF, IL-6 and nitric oxide. Next, topological algorithms were used to identify hub genes in the PPI network. We have found that the therapeutic effects of Methazolamide in AS are mediated at least in part via PTGS2, ESR1, GSK3β, JAK2, and NOS2.The roles of these target proteins in AS were discussed as follows.

### PTGS2

Prostaglandin-Endoperoxide Synthase 2 (PTGS2) is also known as cyclooxygenase 2 (COX-2). Unlike COX-1, PTGS2 is rapidly induced by both inflammatory and mitogenic stimuli, resulting in increased prostaglandin synthesis in inflamed tissues in AS^68^. NSAIDs are the first-line medication for pain and stiffness in AS patients^4^ by inhibiting the synthesis of prostaglandins. The interaction between Methazolamide and PTGS2 may imply the analgesic and anti-inflammatory mechanism of Methazolamide in AS.

### ESR1

The estrogen receptor α, which plays a key role in reproduction and exerts functions in numerous nonreproductive tissues, is encoded by *ESR1*^69^. In 2017, Hyemin Jeong et al. reported that estrogen could attenuate the spondylarthritis manifestations of the SKG arthritis model^70^. Later, they also applied selective estrogen receptor modulator lasofoxifene to suppress joint inflammation and enhance bone mineral density in zymosan-induced SKG mice^71^. Furthermore, oral estrogen therapy in female patients and human chorionic gonadotrophin injections in male patients with AS could result in a moderate clinical improvement^72^. The interaction between Methazolamide and ESR1 may imply the estrogen-related therapeutic strategy of Methazolamide in AS.

### GSK3β

Glycogen synthase kinase-3 beta (GSK3β) is a serine/threonine kinase, which is a negative regulator of glucose homeostasis and a known master regulator for energy metabolism, inflammation, endoplasmic reticulum-stress, mitochondrial dysfunction, and apoptotic pathways^73^. In 2015, Li C et al. reported that miR-29a was significantly down regulated in AS patients. Later, they demonstrated that miR-29a could inhibit TNF-α mediated bone loss mainly by down regulating GSK3β, and promote osteoblast genesis by activating the Wnt/b-catenin pathway^74^. On the other hand, compared with ligament tissues from femoral neck fracture patients, S-L Tang et al. observed the decreased GSK3β level in AS ligament tissue. The declined level of GSK3β was also reported during the osteogenic differentiation process of ligament fibroblast^75^. In summary, the interaction between Methazolamide and GSK3β may imply therapeutic effect of Methazolamide associated with bone loss and ligament calcification in AS.

### JAK2

The Janus kinases (JAKs) are the non-receptor tyrosine kinase^76^. JAK2 is the only AS-associated JAK^63,77^. It was reported that JAK2 was involved in osteoblast differentiation^78^ and inflammation pathways driven by IL-23^79^ in AS. In addition, JAK inhibitors have demonstrated significant efficacy in patients with highly active AS^80^. The interaction between Methazolamide and JAK2 may imply therapeutic effect of Methazolamide through the JAK2 related pathway in AS.

### NOS2

NOS2 is the inducible nitric oxide synthase (iNOS) which is independent of elevated intracellular Ca2 +^81^. In 1999, K E Armour et al. have observed the increased NO production in an animal model of inflammation-induced osteoporosis accompanied by activation of iNOS in the bone marrow space^82^. Later, Dominique Lamarque et al. showed that iNOS activity in duodenum and colon, as well as expression of iNOS protein in lamina propria inflammatory cells, were increased in AS patients compared to controls^83^. Recently, the relationship between NOS2 and AS patients with acute anterior uveitis was confirmed in a genomewide association study^84^. The interaction between Methazolamide and NOS2 may imply NO related anti-inflammatory mechanism of Methazolamide in AS.

Notably, we innovatively intersected the KEGG and GO terms of target genes with the GSEA results of validate dataset (GSE73754) in this study. This method may do favor to elucidate the therapeutic mechanisms of Methazolamide in treating AS more precisely under the guidance of NES and FDR in GSEA. In Supplementary Table S2, the enriched RELA-PTGS2/NOS2 axis contributes to angiogenesis in KEGG small cell lung cancer pathway^85^ (see Supplementary Fig. S1), implying that Methazolamide may inhibit the progression of AS by down-regulating the angiogenesis process^86^. Similarly, Methazolamide may relieve inflammation and ossification by targeting the intersect genes (*GSK3β, JAK2, RELA*)^75,78^ in KEGG chemokine signaling pathway^85^ (see Supplementary Fig. S2). In biological process, Methazolamide may reduce the ossification of AS patients via targeting ALPL and PTGS2 in bone mineralization process.

Molecular docking between the target proteins and Methazolamide were conducted successfully. Molecular dynamics simulations have become a standard tool for the investigation of biomolecules to assess stability and flexibility. Simulations are performed of ever bigger systems using more realistic boundary conditions and better sampling due to longer sampling times to ensure the reliability of molecular docking^87–89^. In this study, re-docking and molecular dynamics simulations were completed to ensure the reliability of molecular docking.

This study explored the pathogenic role of CA1 and the therapeutic mechanism of Methazolamide in AS through the bioinformatics and Network Pharmacological Analysis. Meanwhile, the validate dataset from the GEO database was used to further confirm the therapeutic mechanism of Methazolamide in AS. Nevertheless, in vivo/vitro experiments are still needed to validate these mechanisms and provide more therapeutic targets in AS.

## Conclusion

In summary, CA1 is involved in the specific pathogenic mechanism of AS via one carbon metabolic process, zinc ion bind, transition metal ion binding, hydrolase activity acting on ester bonds, arylesterase activity, carbonate dehydratase activity and nitrogen metabolism pathways. In addition, Methazolamide is reasonable to be the promising drug for treating AS. As a CA inhibitor, Methazolamide may exert therapeutic effects on AS mainly through inflammatory pathways which regulate the production of TNF, IL-6 and nitric oxide. PTGS2, ESR1, GSK3β, JAK2, NOS2 and CA1 were selected as therapeutic targets of Methazolamide in AS. Furthermore, we innovatively intersected the GO/KEGG analyses of target genes with the GSEA results of validate dataset, and found that 18 GO terms and 5 KEGG terms were indicated in the pharmacological mechanism of Methazolamide in AS, involving bone mineralization, angiogenesis, inflammation, and chemokine signaling pathways.

### Supplementary Information

Supplementary Figures.

Supplementary Legends.

Supplementary Information 1.

Supplementary Information 2.

Supplementary Information 3.

Supplementary Information 4.

Supplementary Information 5.

Supplementary Tables.

## Data Availability

The datasets generated and/or analyzed for this study can be found in the NCBI GEO repository https://www.ncbi.nlm.nih.gov/geo/query/acc.cgi?acc=GSE73754^52^.
