# Supplementary material for: Revealing mechanism of Methazolamide for treatment of ankylosing spondylitis based on network pharmacology and GSEA
Source: Sci Rep. 2023 Sep 16;13:15370. doi: 10.1038/s41598-023-42721-x (PMC10505193; doi:10.1038/s41598-023-42721-x)
Supplement: Supplementary file 1 — Supplementary Figures. [file 41598_2023_42721_MOESM1_ESM.pdf]

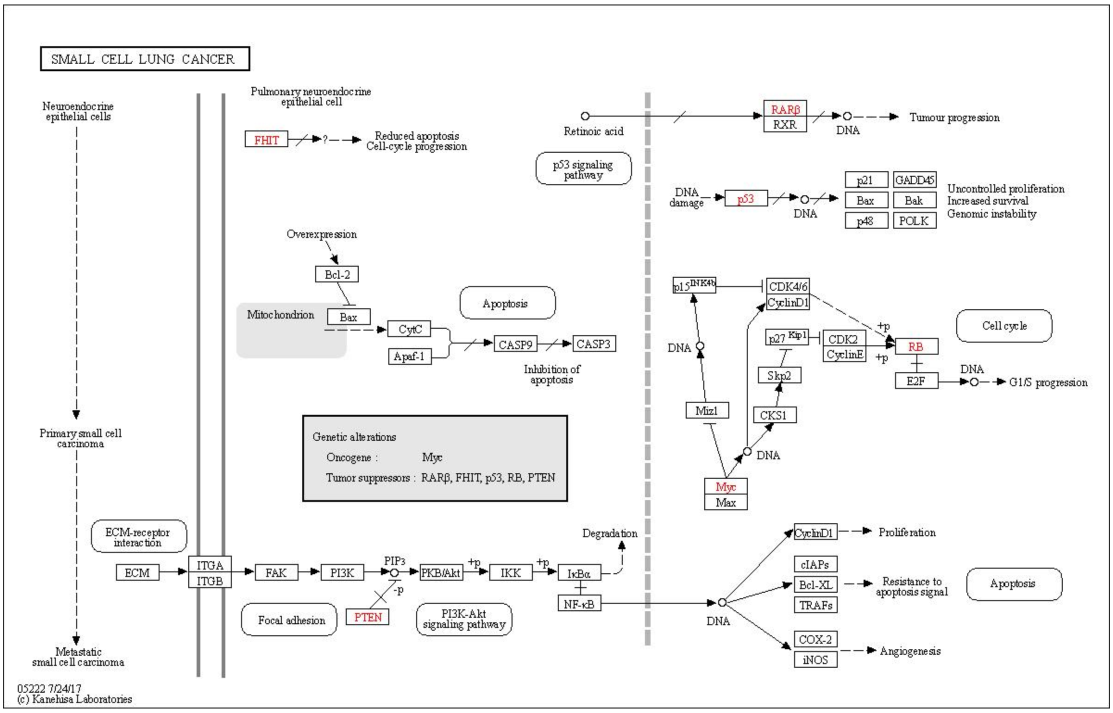

Supplementary Fig. S1. The KEGG small cell lung cancer pathway from the website www.kegg.jp/kegg/kegg1.html.

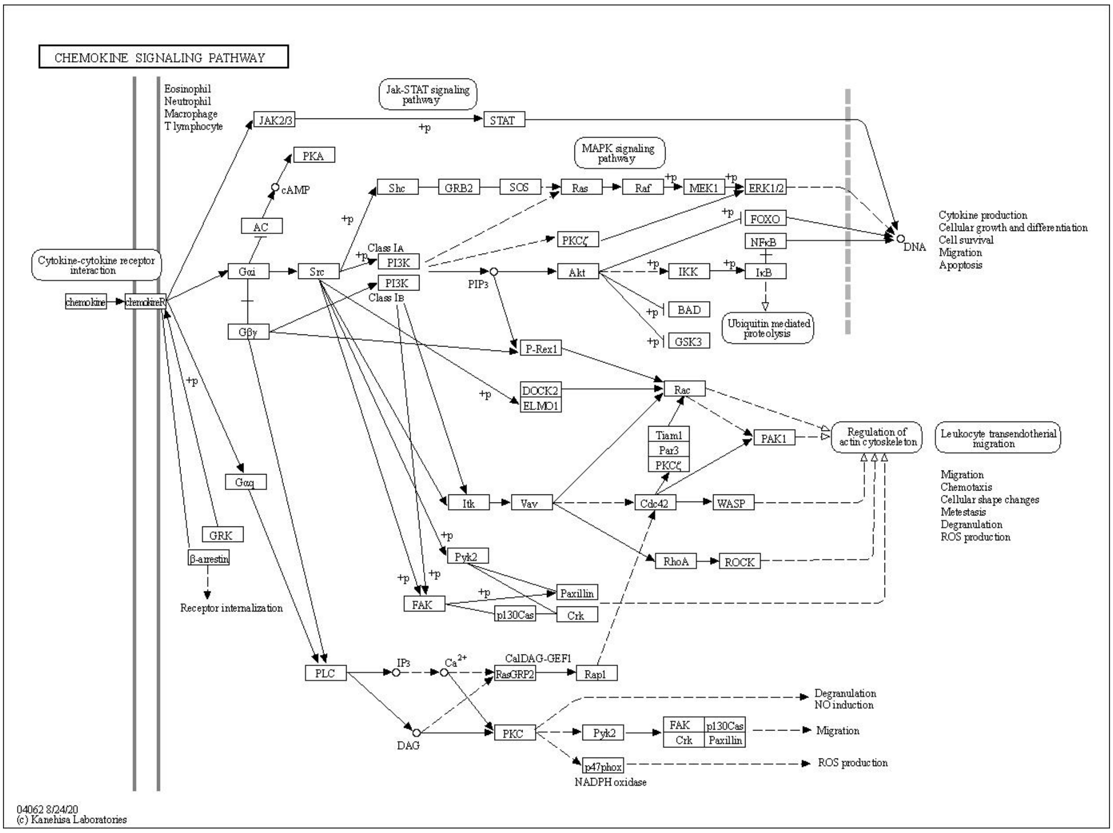

Supplementary Fig. S2. The KEGG chemokine signaling pathway from the website www.kegg.jp/kegg/kegg1.html.
