# Supplementary material for: Revealing mechanism of Methazolamide for treatment of ankylosing spondylitis based on network pharmacology and GSEA
Source: Sci Rep. 2023 Sep 16;13:15370. doi: 10.1038/s41598-023-42721-x (PMC10505193; doi:10.1038/s41598-023-42721-x)
Supplement: Supplementary file 2 — Supplementary Legends. [file 41598_2023_42721_MOESM2_ESM.docx]

Supplementary Spreadsheets legends

Revealing mechanism of Methazolamide for treatment of Ankylosing Spondylitis based on network pharmacology and GSEA

Tao Sun^1†^, Manzhi Wang^2†^, Weiqiang Liang^1^, Ping Gao^1^, Qiang Liu^3^, Xinfeng Yan^1^*

***Correspondence: Xinfeng Yan:** [Yanxinfeng123456@163.com](mailto:Yanxinfeng123456@163.com)

# Supplementary Spreadsheets S1: The normalized original data of GSE73754 obtained from the Gene Expression Omnibus (GEO) database.

- 1. **Supplementary Spreadsheets S2:** The union of AS associated genes obtained from TTD database (3 genes), OMIM database (108 genes) and the highest quartile of genes from GeneCards database (642 genes). Totally 728 AS associated genes were selected after removing the duplicates.
  2. **Supplementary Spreadsheets S3:** The union of Methazolamide target genes obtained from TargetNet database (114 genes) and SwissTargetPrediction database (14 genes). Totally 108 Methazolamide target genes were selected after removing the duplicates.
  3. **Supplementary Spreadsheets S4:** Hub genes identified by four topological analysis algorithms (MCC, MNC, Degree, and EPC) through CytoHubba in the PPI network.
  4. **Supplementary Spreadsheets S5:** The AS associated [rank ordered gene list](file:///F:\\Users\\jianhua\\Documents\\x\\ranked_gene_list_AS_versus_CONTROL_1650418346909.tsv) obtained by GSEA.
