# Supplementary material for: Revealing mechanism of Methazolamide for treatment of ankylosing spondylitis based on network pharmacology and GSEA
Source: Sci Rep. 2023 Sep 16;13:15370. doi: 10.1038/s41598-023-42721-x (PMC10505193; doi:10.1038/s41598-023-42721-x)
Supplement: Supplementary file 8 — Supplementary Tables. [file 41598_2023_42721_MOESM8_ESM.pdf]

Supplementary Table S1. Molecular docking between Methazolamide and target proteins

target gene	interaction type	RESNR	RESTYPE	Distance	DIST_H-A	DIST_D-A
PTGS2	Hydrophobic Interactions	349	VAL	3.76		
	Hydrophobic Interactions	352	LEU	3.55		
	Hydrogen Bonds	120	ARG		2.46	3.28
	Hydrogen Bonds	355	TYR		2.28	2.93
ESR1	Hydrophobic Interactions	525	LEU	3.61		
GSB3B	Hydrophobic Interactions	188	LEU	3.55		
	Hydrogen Bonds	85	LYS		2.27	3.12
	Hydrogen Bonds	200	ASP		2.36	3.18
JAK2	Hydrophobic Interactions	983	LEU	3.88		
	Hydrogen Bonds	858	GLY		3.43	3.97
NOS2	Hydrogen Bonds	263	GLN		3.26	3.78
	Hydrogen Bonds	266	ARG		2.64	3.39
	Hydrogen Bonds	382	ASP		2.33	3.31
	Hydrogen Bonds	388	ARG		2.54	3.32
	Hydrogen Bonds	388	ARG		1.81	2.8
CA1	Hydrogen Bonds	64	HIS		2.11	3.04
	Hydrogen Bonds	94	HIS		2.16	3.03
	Hydrogen Bonds	96	HIS		2.22	3.12

Hydrogen Bonds	119	HIS	2.48	3.37
Hydrogen Bonds	199	THR	2.00	2.73
Hydrogen Bonds	200	HIS	2.43	2.98

RESNR: Number of receptor residue in PDB file. Distance: Distance between interactions carbon atoms. RESTYPE: residue type (amino acid). DIST_H-A: distance between hydrogen and acceptor atoms. DIST_D-A: distance between donor and acceptor atoms. VAL: Valine, LEU: Leucine, ARG: arginase, TYR: tyrosine, LYS: Lysine, ASP: Aspartic acid, GLY: Glycine, GLN: Glutamine, ARG: Arginine, HIS: Histidine, THR: threonine.

Supplementary Table S2. The intersection of KEGG and GO analyses of target genes and GSEA results.

Term	intersect genes	NES	FDR q-val
**KEGG_small cell lung cancer**	**NOS2, PTGS2, RELA**	**1.68**	**0.055**
**CC_glutamatergic synapse**	**GSK3B, HTR2A, JAK2, RELA**	**1.60**	**0.149**
**BP_regulation of synaptic vesicle exocytosis**	**GSK3B, HTR2A**	**1.57**	**0.150**
**KEGG_pathways in cancer**	**GSK3B, NOS2, JAK2, PTGS2, ESR1, RELA**	**1.53**	**0.071**
**BP_positive regulation of jnk cascade**	**RIPK2, TLR9, MMP8**	**1.50**	**0.171**
**CC_caveola**	**HTR2A, JAK2, PTGS2**	**1.50**	**0.171**
**MF_dioxygenase activity**	**PTGS2, PTGS1**	**1.50**	**0.173**
**BP_response to vitamin d**	**ALPL, PTGS2**	**1.46**	**0.197**
**MF_transcription coactivator binding**	**ESR1, RELA**	**1.42**	**0.214**
**BP_bone mineralization**	**ALPL, PTGS2**	**1.41**	**0.221**

**KEGG_neurotrophin signaling pathway**	**GSK3B, RIPK2, RELA**	**1.38**	**0.163**
**BP_negative regulation of protein catabolic process**	**NOS2, RELA**	**1.36**	**0.245**
**KEGG_chemokine signaling pathway**	**GSK3B, JAK2, RELA**	**1.32**	**0.203**
BP_aging	HTR2A, PTGS2, RELA	1.31	0.277
BP_extrinsic apoptotic signaling pathway	GSK3B, JAK2	1.30	0.283
BP_positive regulation of vasoconstriction	HTR2A, PTGS2	1.26	0.315
BP_positive regulation of mapk cascade	TLR9, JAK2, MMP8	1.26	0.318
BP_positive regulation of chemokine production	RIPK2, TLR9	1.18	0.375
BP_regulation of inflammatory response	JAK2, PTGS2, ESR1, RELA	1.10	0.443
BP_response to tumor necrosis factor	JAK2, PTGS2	1.10	0.449
KEGG_nitrogen metabolism	CA1	1.09	0.360
BP_positive regulation of erk1 and erk2 cascade	RIPK2, MIF, HTR2A	0.92	0.658
BP_response to antibiotic	ALPL, JAK2	0.91	0.675

GSEA: Gene Set Enrichment Analysis, GO: Gene Ontology KEGG Kyoto Encyclopedia of Genes and Genomes, CC: cellular component, BP: biological process, MF: molecular function, NES: normalized enrichment score, FDR: false discovery rate.
